# Final results of the real-life observational VICTOR-6 study on metronomic chemotherapy in elderly metastatic breast cancer (MBC) patients

**DOI:** 10.1038/s41598-023-39386-x

**Published:** 2023-07-28

**Authors:** B. Trevisan, F. F. Pepe, I. Vallini, E. Montagna, D. Amoroso, R. Berardi, A. Butera, K. Cagossi, L. Cavanna, M. Ciccarese, S. Cinieri, E. Cretella, E. De Conciliis, A. Febbraro, F. Ferraù, A. Ferzi, A. Baldelli, A. Fontana, A. R. Gambaro, O. Garrone, V. Gebbia, D. Generali, L. Gianni, F. Giovanardi, A. Grassadonia, V. Leonardi, S. Sarti, A. Musolino, M. Nicolini, C. Putzu, F. Riccardi, D. Santini, M. G. Sarobba, M. G. Schintu, G. Scognamiglio, P. Spadaro, C. Taverniti, D. Toniolo, P. Tralongo, A. Turletti, R. Valenza, M. R. Valerio, P. Vici, L. Clivio, V. Torri, M. E. Cazzaniga, The VICTOR Study Group

**Affiliations:** 1 Azienda Ospedaliera San Gerardo, Monza, Italy; 2 Ospedale di Circolo e Fondazione Macchi, Varese, Italy; 3 European Institute of Oncology, Milan, Italy; 4 Ospedale Versilia, Camaiore, Italy; 5 Azienda Ospedaliera Universitaria Ospedali Riuniti, Torrette, Italy; 6 Nuovo Ospedale San Giovanni Di Dio, Florence, Italy; 7 Ospedale Ramazzini, Carpi, Italy; 8 Azienda Ospedaliera Piacenza, Piacenza, Italy; 9 Ospedale Vito Fazzi, Lecce, Italy; 10 Ospedale A. Perrino, Brindisi, Italy; 11 Ospedale Bolzano, Bolzano, Italy; 12 ASL Asti, Asti, Italy; 13 Ospedale S. Cuore di Gesù Fatebenefratelli, Benevento, Italy; 14 Ospedale San Vincenzo, Taormina, Italy; 15 Azienda Ospedaliera Ospedale Civile Di Legnano, Magenta, Italy; 16 Ospedale San Salvatore, Coppito, Italy; 17 Azienda Ospedaliero-Universitaria Pisana, Pisa, Italy; 18 ASST Fatebenefratelli Sacco, Milan, Italy; 19 Fondazione IRCCS Ca’ Granda Ospedale Maggiore Policlinico, Milan, Italy; 20 Ospedale La Maddalena, Palermo, Italy; 21 Istituti Ospitalieri Cremona, Cremona, Italy; 22 Ospedale Infermi, Rimini, Italy; 23 AUSL della Romagna, Emilia-Romagna, Italy; 24 Ospedale SS Annunziata, Taranto, Italy; 25 Ospedale Civico, Palermo, Italy; 26 IRCCS Istituto Scientifico Romagnolo per lo Studio e la Cura dei Tumori, Meldola, Italy; 27 Ospedale di Parma, Parma, Italy; 28 Azienda Ospedaliera-Universitaria, Sassari, Italy; 29 Ospedale Antonio Cardarelli, Naples, Italy; 30 Università Campus Bio-Medico, RomE, Italy; 31 Ospedale San Francesco, Nuoro, Italy; 32 Ospedale Giovanni Paolo II, Lecce, Italy; 33 Ospedale Valduce, Como, Italy; 34 Casa di Cura Villa Salus-Messina, Messina, Italy; 35 Ospedale Molinette, Turin, Italy; 36 Ospedale di Circolo, Rho, Italy; 37 Ospedale Umberto I, Rome, Italy; 38 Ospedale Martini, Torino, Italy; 39 P.O. Vittorio Emanuele, Gela, Italy; 40 A.O.U. Policlinico Paolo Giaccone, Palermo, Italy; 41 INT Regina Elena, Rome, Italy; 42 IRCCS Mario Negri Institute of Pharmacological Research, Milan, Italy

**Keywords:** Cancer, Breast cancer

## Abstract

Nowadays, treatment of metastatic breast cancer (MBC) has been enriched with novel therapeutical strategies. Metronomic chemotherapy (mCHT) is a continuous and frequent administration of chemotherapy at a lower dose and so whit less toxicity. Thus, this strategy could be attractive for elderly MBC patients. Aim of this analysis is to provide insights into mCHT’s activity in a real-life setting of elderly MBC patients. Data of patients ≥ 75 years old included in VICTOR-6 study were analyzed. VICTOR-6 is a multicentre, Italian, retrospective study, which collected data on mCHT in MBC patients treated between 2011 and 2016. A total of 112 patients were included. At the beginning of mCHT, median age was 81 years (75–98) and in 33% of the patients mCHT was the first line choice. Overall Response Rate (ORR) and Disease Control Rate (DCR) were 27.9% and 79.3%, respectively. Median PFS ranged between 7.6 and 9.1 months, OS between 14.1 and 18.5 months. The most relevant toxicity was the hematological one (24.1%); severe toxicity (grade 3–4) ranged from 0.9% for skin toxicity up to 8% for hematologic one. This is a large study about mCHT in elderly MBC patients, providing insights to be further investigated in this subgroup of frail patients.

## Introduction

Over the last few years, the treatment of metastatic breast cancer (MBC) has been enriched with many new strategies in terms of chemotherapy, endocrine therapy and targeted drugs. This has resulted in a significant improvement in both Progression-Free (PFS) and Overall Survival (OS) in all MBC populations, mainly due to the availability of immune check point inhibitors in TNBC patients, new drug-conjugated antibodies in HER2+ ones, and Cycline-Dependent Kinase inhibtors in Hormone-Receptor positive (HR+) Human Epidermal Growth Factor Receptor negative (HER2−) patients^1^.

The goal of MBC treatment remains the delay of tumor progression, without negatively impacting patients' quality of life^2^. This has led to the research and the discovery of alternative methods of administering chemotherapeutic agents. In this context, metronomic chemotherapy (mCHT) refers to a continuous and frequent administration of chemotherapy drugs at a lower dose than the maximum tolerated dose (MTD) used in the conventional regimens^3,4^, which leads to less adverse events (AE) and continuative use of therapy without long drug-free intervals.

Several studies have confirmed that mCHT can also be considered a "multi-target" therapy carrying out a triple action^5^: cytostatic effect mediated by direct action on tumor cells^6^, inhibition of angiogenesis^7^ and immunomodulation on the tumor microenvironment, in particular by suppression of Treg lymphocytes and promotion of dendritic cells maturation^8,9^.

Since the 2000s, several clinical trials, mainly phase II, have been developed to evaluate the efficacy of different chemotherapy agents administered with a metronomic schedule^10–15^.

Given the preclinical and clinical evidence showing efficacy of mCHT in MBC, this has been included as treatment option in ABC-ESMO guidelines since 2017^16^.

A meta-analysis of 22 mCHT studies reported an Overall Response Rate (ORR) 34.1% and a clinical benefit rate (CBR) of 55.6%, PFS of 6 months and OS of 12–24 months, grade 3–4 AE in only 29.5% of the patients^15^.

Considering the good toxicity profile, the possibility to maintain quality of life and the minimal requirement for monitoring of blood chemistry tests, this strategy could be attractive for elderly or frail MBC patients, who frequently have multiple comorbidities, can develop a clinical decline, and have adverse drug events^2,17^. Based on different data regarding physical activity and functional independence, the current definition of elderly (65 years over) has been changed to those over 75 years^19^. Elderly patients are usually not included in randomized clinical trials, so age represents itself an important barrier to derive data in this population of patients.

This patients’ group is often not included in clinical trials, mainly due to their frailty and the presence of comorbidities that can expose them to a greater risk of developing toxicity. There is no gold standard of treatment in these patients and the efficacy data of chemotherapy in this population are limited ^1919^Very often, this subgroup of patients receives non-standard treatment options and dose reductions. This might compromise the treatments’ efficacy and lead to a worse outcome^20^.

VICTOR-6 was a multicenter retrospective cohort study, which collected data of 584 MBC patients who received mCHT between January 2011 and December 2016 at 43 Italian Oncology sites. Aim of the study was to describe the use of mCHT to collect data regarding the different type and regimens of metronomic drugs administered, their efficacy and safety. Pre-planned analyses included description of efficacy and safety in TNBC and elderly populations. Here we report data regarding the population of elderly patients (≥ 75 years old) enrolled in the abovementioned trial^24^.

## Materials and methods

### Study population

**Figure 1:**
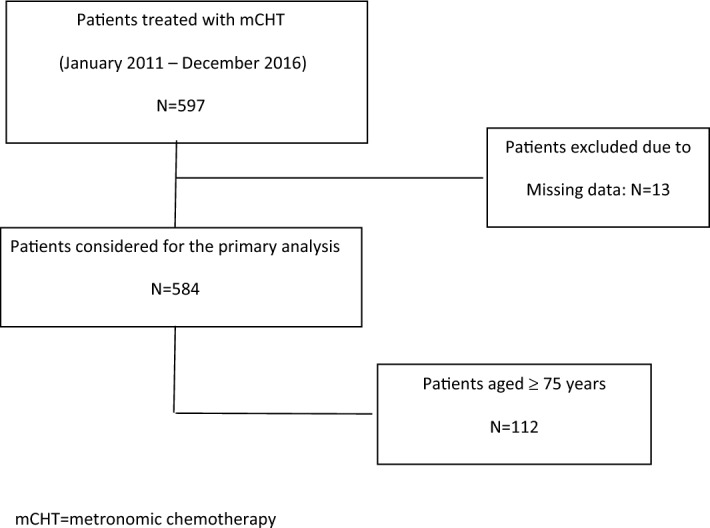
CONSORT flow chart.

Centers selection and Hospital characteristics have already been reported in the main paper^24^. Briefly, the centers selected usually treat more than 150 new cases of breast cancer per year and can be considered representative of the national population as a whole.For the present analysis, we identified all the patients aged 75 years or more enrolled in the VICTOR-6 study (Fig. 1). As Phase 1 Research Center was the coordinating center, the trial was approved by Comitato Etico Brianza. If still alive at the moment of data collection, all patients provided written informed consent.

Data was collected into an electronic database. Patients’ and tumors’ characteristics, such as age and stage at diagnosis, breast cancer biology (e.g. histology, HR and HER2 status), metastatic sites, and previous medical treatments were collected. Efficacy outcomes were reported as best responses to the metronomic treatment.

Study design was fully described in the previously published main paper^24^.

For the present analysis, the eligible patients were female, ≥ 75 years, with documented locally advanced or MBC, previously treated or not with other drugs for the metastatic disease, for whom mCHT was chosen by the physician, according to the clinical situation of the patient. All patients who received at least one dose of mCHT were considered eligible. Other inclusion criteria were HER2-negative disease (IHC 0 and 1 or IHC 2, confirmed as FISH negative), measurable or evaluable lesions and availability of all requested data.

We confirm that all methods were performed in accordance with the relevant guidelines and regulations.

### End points

The primary endpoint of this analysis was to describe disease characteristics of elderly women who received a mCHT regimen at any time of their metastatic history. Secondary end points were overall response rate (ORR) and disease control rate (DCR), defined as the sum of Complete + Partial Responses + Stable Disease, according to the type of mCHT, Overall Survival (OS), according to the type of mCHT regimen and the line of treatment and toxicity. As in the main paper, all the surviving patients who did not have a progression of their disease were censored in October 2017.

### Statistical analysis

Demographic data, patients’ and diseases’ baseline characteristics, treatment information were summarized with standard summary statistics: standard deviation and range for continuous data, relative and absolute frequencies for categorical data. These variables' relationship with response were analyzed byMantel–Haenzel test. Time to event analysis was described by Kaplan–Meier approach.

An association with baseline characteristic was analyzed by stratified log-rank test and proportional hazard model. Univariate and multivariate logistic analyses were used to estimate the association of basal characteristics and treatment with response. Odds ratio and relative 95% confidence interval (CI) were used as summary statistics. The number of patients was calculated to obtain a quite precise description of chosen statistics and a good fit with the Cox model. The data were statistically analyzed using SAS version 8 (SAS Institute Inc., Cary, NC). Considering the study design, no statistical comparison was allowed between the population aged ≥ 75 years and the younger one.

## Results

Data extraction from the VICTOR-6 database with the age cut-off at 75 years identified 112 patients, who represent 19.2% of the whole population.

Main tumor characteristics at primary diagnosis were ductal histology (76.7%), pT2 stage (30.8%) and grading G3 (44.7%). Seventy-eight patients (69.6%) had Luminal-like subtype tumors (ER+ /PR+ ; N = 56, 50%, ER+ /PR− or ER− /PR+ ; N = 22, 19.6% ), 34 (30.4%) patients had triple negative breast cancer (TNBC) and only one patient had an unknown receptor status. A quarter of the patients were metastatic de novo.

Median Disease-Free Interval was 24 months (0–610).

Median age at first metastatic diagnosis was 79 years (75–98), and the main involved sites were bone (46.4%), lung (18.8%), and liver (16.9%). Forty-four patients (39.6%) received chemotherapy, endocrine treatment (55.0%) or both (27.0%) as first treatment.

At the beginning of mCHT, median age was 81 years (75–98) and most patients had an ECOG PS of 0 (40.2%) or 1 (46.4%). The majority had ≤ 2 metastatic sites (97, 86.6%) and main localizations were at bone (56.3%), liver (26.8%) and lung (22.3%).

In 33% of the patients (N = 37), mCHT was the first line choice. Most patients had previously received standard chemotherapy, alone (16, 14.3%), or followed by endocrine therapy (24, 21.4%). Previous therapies were mainly anthracycline- and taxane-based regimens (9.8% and 12.5% respectively). Table 1 summarizes disease characteristics at the time of mCHT start in both elderly and younger populations.

**Table 1:** Patients and tumor characteristics at mCHT start.

Characteristics	≥ 75 years (%) N = 112	< 75 years (%) N = 472
HR status (at primary diagnosis)		
ER+ /PgR+	56 (50.0)	318 (67.4)
ER+ /PgR− or ER− /PgR+	22 (19.6)	91 (19.3)
TNBC	34 (30.4)	63 (13.3)
PS		
0	45 (40.2)	300( 63.6)
1	52 (46.4)	138 (29.2)
2	14 (12.5)	28 (5.9)
3	1 (–)	4 (–)
Not available	0	2
Metastatic sites		
Bone	63 (56.3)	333 (70.6)
Lung	25 (22.3)	157 (33.3)
Liver	30 (26.8)	199 (42.2)
Soft tissue	22 (19.6)	88 (18.7)
Others	35 (31.3)	181 (38.3)
Number of metastatic sites		
1	59 (52.7)	147 (31.1)
2	38 (33.9)	200 (42.4)
≥ 3	12 (10.7)	118 (25.0)
NA	3 (2.7)	7 (1.4)
Number of treatments before mCHT		
0	37 (33.0)	74 (15.7)
1	33 (29.5)	84 (17.8)
2	17 (15.2)	106 (22.5)
≥ 3	25 (22.3)	208 (44.1)

**Figure 2:**
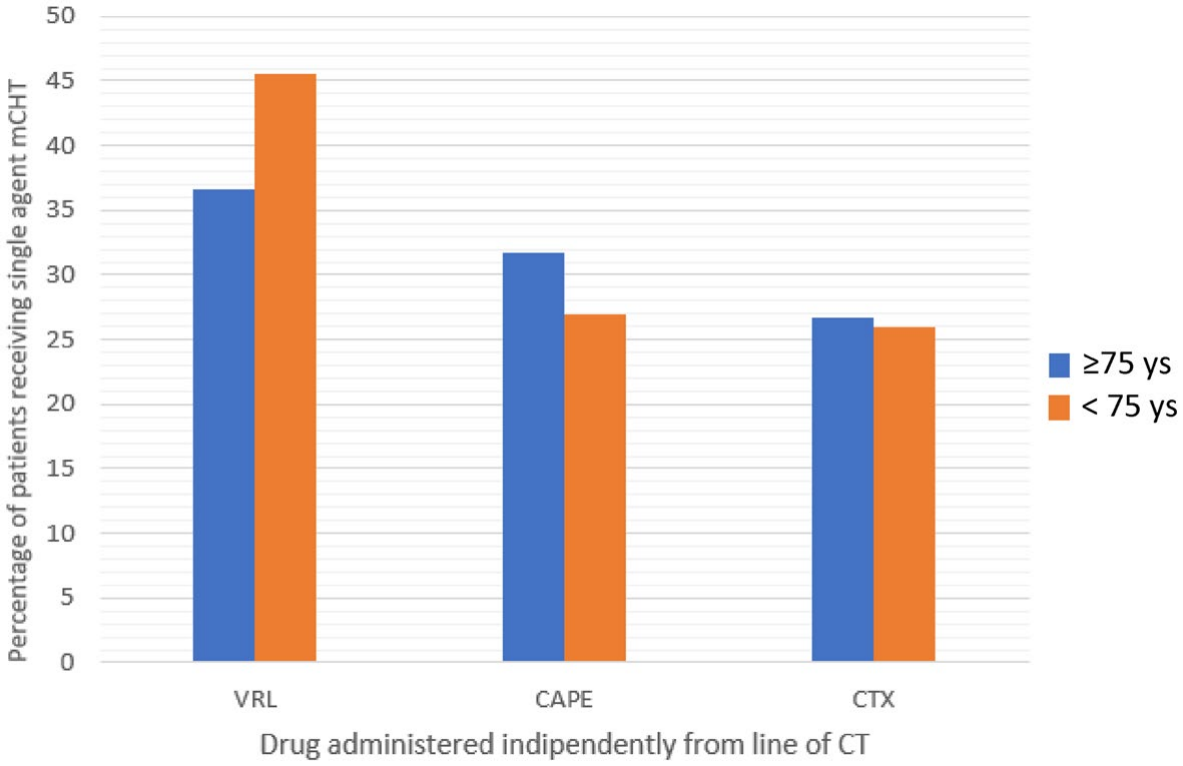
Percentages of patients receiving single agents mCHT.

Most patients were treated with Vinorelbine (VRL)-based regimens (46, 41.1%), followed by Capecitabine (CAPE)-based (33, 29.5%), Cyclophosphamide (CTX)-based (28, 25%) and Methotrexate (MTX)-based (5, 4.5%) regimens; 90.2% of the patients (N = 101) received a single agent mCHT: 36.7% of them a VRL-based regimen, 31.7% a CAPE-based, 26.7% a CTX-based and 5% a MTX-based. In Fig. 2, Single Agent mCHT regimens in the two populations (≥ 75 and < 75 years) are presented, to descriptively highlight similarities and differences.

One-hundred eleven patients were assessable for the evaluation of clinical activity; one patient was lost before performing imaging procedures. Overall Response Rate (ORR) and DCR were 27.9% and 79.3%.

The highest ORRs were observed for the VRL-based regimens (48.3%), especially in the first line setting, followed by CAPE-based treatments (25.8%).

ORR according to the line of treatment and the type of mCHT in elderly patients is described in Table 2, whereas Tables 1S and 2S describe ORRs in the elderly and younger populations.

**Table 2:** ORR according to the line of treatment and the type of mCHT in elderly patients.

ORR	%
Overall (N = 111)	27.9
1st-line (N = 73)	35.2
VRL-based (N = 30)	46.7
CAPE-based (N = 20)	30.0
CTX-based (N = 18)	20.0
MTX-based (N = 5)	40.0
2nd-line (N = 29)	13.8
CAPE-based (N = 12)	41.4
VRL-based (N = 11)	37.9
CTX-based (N = 2)	
3rd-line (N = 5)	20.0
4th-line (N = 4)	25.0

The most relevant toxicity was the hematological one (24.1%, any grade), followed by gastrointestinal, mainly nausea/vomiting (13.4%), diarrhea (12.5%) and impaired liver function (4.5%); other non-hematologic toxicities were asthenia and skin reactions, which were reported in 11.6% and 7.1% of the patients, respectively.

Regarding severe toxicities, Grade 3–4 hematological one was reported in 8% of the cases, G3-4 nausea/vomiting in 2.7% and severe diarrhea in 1.8%. Details regarding toxicity are summarized in Table 3.

**Table 3:** Safety details according to patients’ age.

Toxicity	≥ 75 years, n (%)	< 75 years, n (%)
Tot patients	N = 112	N = 472
Hematologic	27 (24.1)	93 (19.7)
G1-2	18 (16.1)	66 (13.9)
G3-4	9 (8.03)	27 (5.7)
Nausea/vomiting	15 (13.4)	90 (19.1)
G1-2	12 (10.7)	81 (17.2)
G3-4	3 (2.7)	9 (1.9)
Diarrhea	14 (12.5)	65 (13.8)
G1-2	12 (10.7)	61 (12.9)
G3-4	2 (1.8)	4 (0.8)
Fatigue	13 (11.6)	53 (11.2)
G1-2	11 (9.8)	50 (10.6)
G3-4	2 (1.8)	3 (0.6)
Cutaneous	8 (7.1)	60 (12.7)
G1-2	7 (6.3)	47 (9.9)
G3-4	1 (0.9)	13 (2.7)
Hepatic	5 (4.5)	38 (8.0)
G1-2	3 (2.7)	32 (6.8)
G3-4	2 (1.8)	6 (1.3)
Other toxicity	11 (9.8)	61 (12.9)
G1-2	7 (63.6)	55 (90.2)
G3-4	4 (36.4)	6 (9.8)

Discontinuation due to adverse events was observed in only 13 patients (11.6%).

Median PFS was 9.1 months (95% CI 6.8–17.5) for CAPE-based therapy, 8.8 months (95% CI 7–11.8) for CTX-based and 7.6 months (95% CI 6.2–12.1) for VRL-based ones.

**Figure 3:**
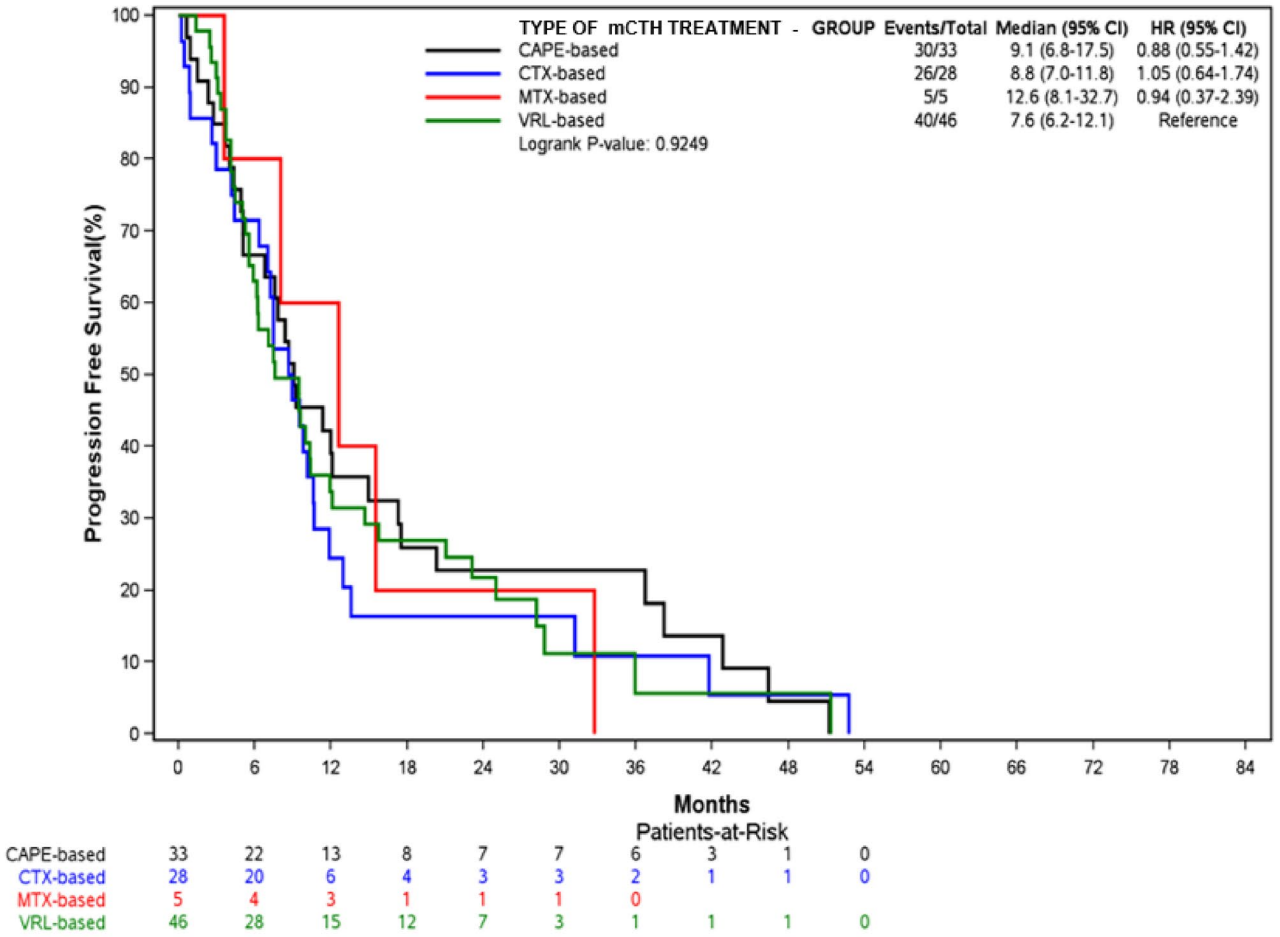
PFS by type of mCHT treatment.

**Figure 4:**
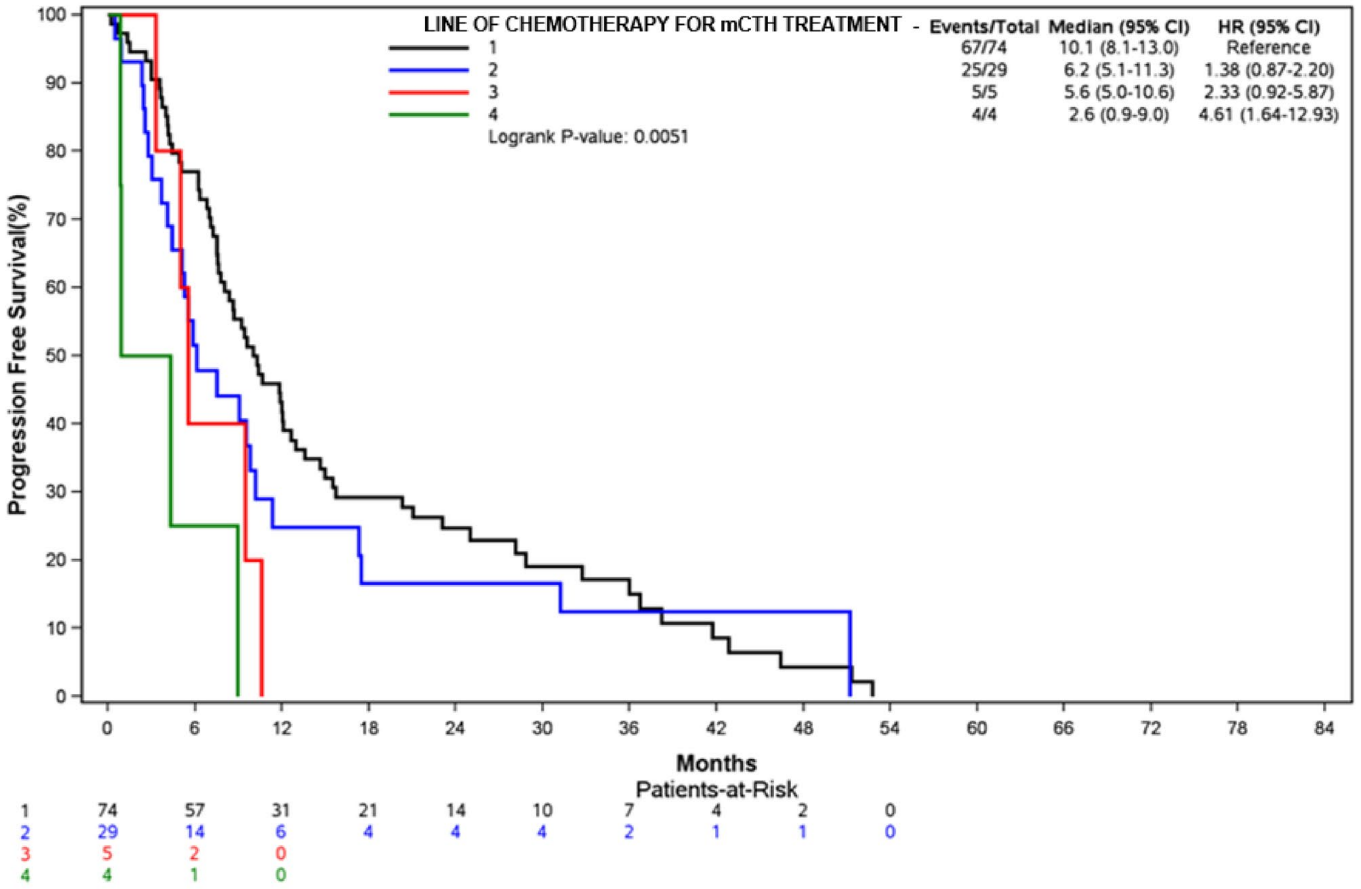
PFS according to the line of mCHT treatment.

The longest median PFS was observed when mCHT was administered in first-line setting (10.1 months, 95% CI 8.1–13) versus 6.2 months (95% CI 5.1–11.3) and 5.6 months (95% CI 5–10.6) for second and third lines, up to 2.6 months (95% CI 0.9–9) for subsequent lines. Kaplan–Meyer estimated PFS according to the type and the line of mCHT are reported in Figs. 3 and 4.

Median OS was 18.1 months (95% CI 15–28.8) for VRL-based regimens, 16.4 months (95% CI 12.9–38.3) for CAPE-based and 14.5 months (95% CI 10.6–51.3) for CTX-based mCHT. A better OS was achieved in relation to the line of treatment: 23.8 months (95% CI 15.4–28.8) versus 15.5 months (95% CI 11.5–41.5) versus 10.6 months (95% CI 5–17.4) in 1st, 2nd and 3rd line, respectively. Figures 1S and 2S report Kaplan-Meyer estimated curves for OS according to the type of mCHT and the line of mCHT, respectively.

## Discussion

To our knowledge, this is the largest retrospective analysis focusing on the treatment of very elderly MBC patients receiving mCTH in a real life setting. Considering that, for cancer-related deaths, more than 35% of the women who died from breast cancer are 75 years old or older and 15% are aged 85 years or older, this over-representation of elderly women in the breast cancer population is projected to dramatically increase within the next two decades^21^. Thus, it is important to derive data, at least from retrospective, observational studies, to provide a basis on which a clinician could consider active treatment in an elderly woman despite age. Generally, data on palliative chemotherapy (CHT) in very elderly patients are rare: in a recently published paper, Overgaauw et Al. retrieved clinical records of patients older than 75 years who received first-line chemotherapy in 2 large teaching hospitals in The Netherlands between 2000 and 2014, finding only 54 evaluable patients^22^ Other different studies on palliative therapy in very elderly patients reported similar difficulties in enrolling this population^23^.

In our series, a quarter of the patients had an advanced disease at the time of the diagnosis. Usually, data report an incidence of the de novo disease in 3%-6% of all new breast cancer diagnoses in high-income Countries and this incidence has not been reduced along decades of screening programs^25^. It is possible that the high incidence of metastatic tumors at diagnosis observed in our population is linked to the point of observation of the study: given that many oncologists consider metronomic therapy free from important complications in terms of toxicity, they could have been more prone to treat a category of patients usually not candidate to active therapies. However, it is well described in geriatric oncology literature that there are different tumor-extrinsic features between older and young age groups and their impact on treatment efficacy and outcome.

Even considering the impossibility to make comparisons between elderly and young populations, one of the most intriguing results is the similar ORR observed: 27.9% and 25.3%, respectively, despite the predominant use of single-agent mCHT in the cohort aged ≥ 75 years. These data confirm that from one side mCHT should not be considered only as a palliative treatment for frail patients and, from the other side, that these patients should not be excluded from therapies only due to their age. In our study, mCHT was administered as first-line treatment in 33% of the patients, twice the percentage reported for the younger population: this may be due to the well-known better tolerability and the lower number of hospital accesses required by this regimen. Our results reflect the recommendations provided by International Society of Geriatric Oncology (SIOG), to be less impacting on QoL, give less toxicity with the most efficacy possible^28^.

In clinical practice, monotherapy is generally preferred over combination chemotherapy since multi-drug regimens are usually associated with increased toxicity and little survival benefit compared with the consecutive administration of single drugs^29^. Our data reflect this choice: in the elderly population a single agent mCHT was chosen in most patients, while younger patients were most likely to be treated with doublets (VRL + CAPE), or triplets (VRL + CAPE + CTX).

Elderly patients are often undertreated when chemotherapy is needed due to disease characteristics or HR loss^21^: drugs with safer profiles are to be preferred, like weekly taxane regimens, capecitabine, vinorelbine or gemcitabine. However, these drugs are often used in the elderly population by adopting dose reductions or other schedule adjustments, but data regarding the real efficacy of reduced/adjusted schedules are lacking^29^. It is also well known that elderly patients are less likely to receive chemotherapy than the younger ones, based on many reasons including concern for toxicity in the frail, comorbidities, and older age itself^30,31^. In this context, mCHT could become an important option of treatment considering that age is not a barrier for a full-dose administration of mCHT.

Different studies have evaluated mCHT approach in elderly MBC patients, reporting results similar to those observed in the real-life VICTOR-6 study.

Addeo et Al., in a Phase 2 trial, treated 34 MBC elderly patients (median age 75 years) with VRL, a vinca alkaloid, at the dose of 70 mg/m^2^ three days a week for 21 days in a cycle of 28. These Authors reported an ORR of 38%, a CBR of 68%, a median PFS of 7.7 months and mOS of 15.9 months. Grade >  = 3 hematologic toxicity occurred only in 6% of patients, while non-haematological toxicity was represented by nausea and vomiting in 44% and 21%, diarrhea and fatigue in 21% and 12% respectively^32^.

De Iulis et Al. a single agent metronomic schedule of VRL (30 mg/die every other day) in 32 MBC women with a median age of 76 years. CBR was about 50%, median PFS of 9.2 months; no Grade 3–4 adverse events (AE) were reported, with a very good preservation of quality of life^2^.

In the VICTOR-1 study, Cazzaniga et Al., evaluated the efficacy and safety of VRL (40 mg thrice a week) and CAPE (500 mg thrice a week) in 32 elderly MBC patients (> 70 years). They reported an ORR of 33%, a CBR of 67% and a median Time To Progression (TTP) of 10.5 months (range 1–40) It was also shown a grade 3–4 AE reduction, confirming a better tolerability of metronomic administration^33^.

Toxicity remains one of the most important concern for CHT administration in elderly patients, in whom a particular attention should be paid to supportive care, since neutropenia is frequently developed, also due to the poor functional bone marrow reserve^29^. In our study, the most common toxicity was the hematological one, occurring in 24.1% of patients ≥ 75 yo (mostly grade 1–2). Even if globally very low, our data suggest that elderly patients should deserve a closer control in comparison to younger patients, in particular regarding blood monitoring and gastro-intestinal effects, which are known to be associated with a higher risk of hospitalization^24^ Generally, mCHT treatment in the older population remains very well tolerated: in our series, severe toxicity (grade 3–4) ranged from 0.9% for skin toxicity up to 8% for hematologic one.

Potthoff et al. in their NABUCCO study investigated AEs associated with Nab-Paclitaxel administration in young (< 70 years) and old (≥ 70 years) cohort of patients: they found no substantial differences in terms of AE (any grade) development between the two subgroups (83.3% of the younger, 86.0% of elderly patients). In particular, peripheral sensitive neuropathy was the most common side effect in both the cohorts (younger 39.7% and elderly 37.4%)^34^. In our study this trend has been confirmed too. Indeed AE, except hematological toxicity, had the same incidence as in older as in younger population, e.g. Diarrhea (any grade) 12.5% versus 13.8% or fatigue (any grade) 11.6 versus 11.2%, respectively.

In the last few years, newer studies looking at the impact of the toxicity to activities of daily living (ADL) have been published^35^. Unfortunately, due to the retrospective design, ADL was not available in clinical records and this represent the major limit of our analysis^30^.

The study has some limitations: a comprehensive geriatric assessment was not done, due to the retrospective collection of the cases. We are aware that, with the advent of artificial intelligence and natural language processing tools, it has become possible to extract this information from EHRs in recent years: however, in Italy there are some limitations in doing that, mainly the persistent use of paper records, which makes the subsequent extrapolation of health data almost impossible.

Patients’ comorbidities too have not been collected, even if it is well known that they may influence the development of toxicities and may affect patients’ quality of life. Despite these limits, we believe that the analysis of the subgroup of elderly patients treated with mCHT could be of value for clinical practice, providing unique information regarding the efficacy of this type of administration.

In the future, clinical trials focusing on elderly cancer patients to determine the best mCHT regimen and the impact on patients' quality of life are strongly recommended.

## Conclusion

To our knowledge this is the largest analysis about mCHT in elderly MBC patients in a real-life setting. Considering that the number of elderly patients affected by MBC will inevitably increase in the next few years, we believe that our analysis could help with the best therapeutic choice in this subgroup of patients. We also hope that it could be of inspiration to conduct further specific studies that will include necessary evaluations, such as ancient patients’ comorbidities and a geriatric assessment.

## Supplementary Information

Supplementary Figure S1.

Supplementary Figure S2.

Supplementary Table S1.

Supplementary Table S2.

## Data Availability

The data used and/or analyzed in the current study are available from the corresponding author on reasonable request.
