# Supplementary figures and images for: Final results of the real-life observational VICTOR-6 study on metronomic chemotherapy in elderly metastatic breast cancer (MBC) patients
Source: Sci Rep. 2023 Jul 28;13:12255. doi: 10.1038/s41598-023-39386-x (PMC10382472; doi:10.1038/s41598-023-39386-x)

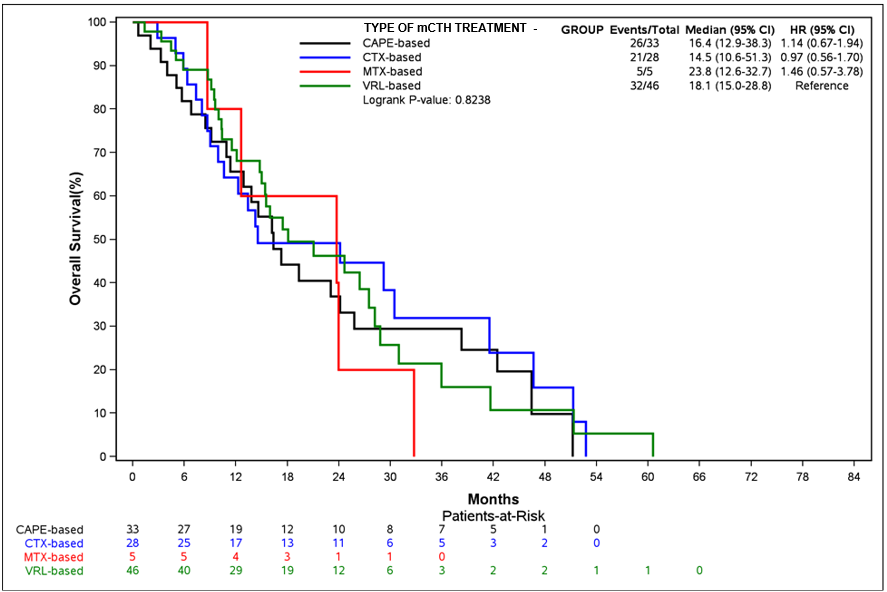


Figure 1S - OS by type of mCHT treatment

Supplement: Supplementary file 1 — Supplementary Figure S1. [file 41598_2023_39386_MOESM1_ESM.docx]

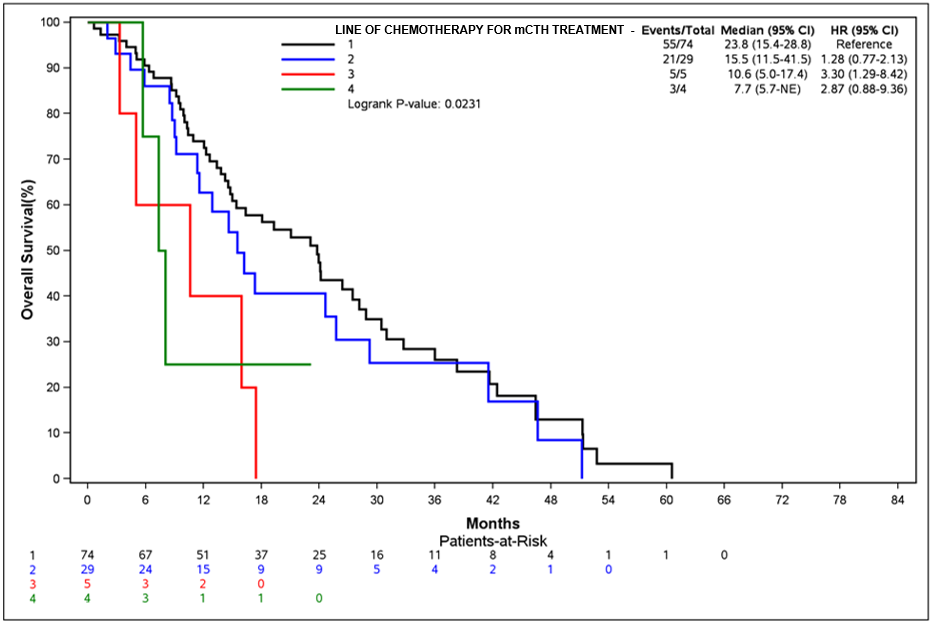


Figure 2S - OS according to the line of mCHT treatment

Supplement: Supplementary file 2 — Supplementary Figure S2. [file 41598_2023_39386_MOESM2_ESM.docx]
