# Supplementary material for: Final results of the real-life observational VICTOR-6 study on metronomic chemotherapy in elderly metastatic breast cancer (MBC) patients
Source: Sci Rep. 2023 Jul 28;13:12255. doi: 10.1038/s41598-023-39386-x (PMC10382472; doi:10.1038/s41598-023-39386-x)
Supplement: Supplementary file 3 — Supplementary Table S1. [file 41598_2023_39386_MOESM3_ESM.docx]

Table 1S - ORR according to the line of therapy in patients aged ≥ 75 vs < 75 years

|  | **≥ 75 ys**  **n/N (%)** | **< 75 ys**  **n/N (%)** |
| --- | --- | --- |
| ORR Overall | 31/111 (27.9) | 118/467 (25.3) |
| 1^st^ line | 25/73 (34.2) | 63/187 ( 33.7) |
| 2^nd^ line | 4/29 (13.8) | 35/144 (24.3) |
| 3^rd^ line | 1/5 (20) | 15/72 (20.8) |
| 4^th^ line | 1/4 (25) | 5/64 (7.8) |
