# Supplementary material for: Final results of the real-life observational VICTOR-6 study on metronomic chemotherapy in elderly metastatic breast cancer (MBC) patients
Source: Sci Rep. 2023 Jul 28;13:12255. doi: 10.1038/s41598-023-39386-x (PMC10382472; doi:10.1038/s41598-023-39386-x)
Supplement: Supplementary file 4 — Supplementary Table S2. [file 41598_2023_39386_MOESM4_ESM.docx]

Table 2S - DCR according to the line of therapy in patients aged ≥ 75 vs < 75 years

|  | ≥ 75 ys  n/N (%) | < 75 ys  n/N (%) |
| --- | --- | --- |
| DCR Overall | 88/111 (79.3) | 342/476 (71.8) |
| 1st line | 60/74 (81.1) | 152/186 (81.7) |
| 2nd line | 22/29 (75.9) | 113/144 (78.5) |
| 3rd line | 4/5 (80.0) | 42/72 (58.3) |
| 4th line | 2/4 (50.0) | 35/64 (54.7) |
